# High-Fructose, High-Fat Diet Alters Muscle Composition and Fuel Utilization in a Juvenile Iberian Pig Model of Non-Alcoholic Fatty Liver Disease

**DOI:** 10.3390/nu13124195

**Published:** 2021-11-23

**Authors:** Heather C. Spooner, Stefani A. Derrick, Magdalena Maj, Rodrigo Manjarín, Gabriella V. Hernandez, Deepali S. Tailor, Parisa S. Bastani, Rob K. Fanter, Marta L. Fiorotto, Douglas G. Burrin, Michael R. La Frano, Angelos K. Sikalidis, Jason M. Blank

**Affiliations:** 1 Department of Biological Sciences, California Polytechnic State University, San Luis Obispo, CA 93407, USA; heather.c.spooner@gmail.com (H.C.S.); mmaj@calpoly.edu (M.M.); deepalistailor@gmail.com (D.S.T.); pbastani101@gmail.com (P.S.B.); 2 Department of Food Science and Nutrition, California Polytechnic State University, San Luis Obispo, CA 93407, USA; sderrick@calpoly.edu (S.A.D.); mlafrano@calpoly.edu (M.R.L.F.); asikalid@calpoly.edu (A.K.S.); 3 Department of Animal Sciences, California Polytechnic State University, San Luis Obispo, CA 93407, USA; rmanjari@calpoly.edu (R.M.); gabiiivh@gmail.com (G.V.H.); 4 College of Agriculture Food and Environmental Sciences, California Polytechnic State University, San Luis Obispo, CA 93407, USA; rfanter@calpoly.edu; 5 Cal Poly Metabolomics Service Center, California Polytechnic State University, San Luis Obispo, CA 93407, USA; 6 United States Department of Agriculture-Agricultural Research Services, Children’s Nutrition Research Center, Department of Pediatrics, Division of Gastroenterology, Hepatology and Nutrition, Baylor College of Medicine, Houston, TX 77030, USA; martaf@bcm.edu (M.L.F.); dburrin@bcm.edu (D.G.B.); 7 Center for Health Research, California Polytechnic State University, San Luis Obispo, CA 93407, USA

**Keywords:** NAFLD, Iberian pig, fuel utilization, western diet, high-fructose diet, high-fat diet, muscle, pediatric, skeletal muscle, probiotics

## Abstract

Non-alcoholic fatty liver disease (NAFLD) is a serious metabolic condition affecting millions of people worldwide. A “Western-style diet” has been shown to induce pediatric NAFLD with the potential disruption of skeletal muscle composition and metabolism. To determine the in vivo effect of a “Western-style diet” on pediatric skeletal muscle fiber type and fuel utilization, 28 juvenile Iberian pigs were fed either a control diet (CON) or a high-fructose, high-fat diet (HFF), with or without probiotic supplementation, for 10 weeks. The HFF diets increased the total triacylglycerol content of muscle tissue but decreased intramyocellular lipid (IMCL) content and the number of type I (slow oxidative) muscle fibers. HFF diets induced autophagy as assessed by LC3I and LC3II, and inflammation, as assessed by IL-1α. No differences in body composition were observed, and there was no change in insulin sensitivity, but HFF diets increased several plasma acylcarnitines and decreased expression of lipid oxidation regulators *PGC1α* and *CPT1*, suggesting disruption of skeletal muscle metabolism. Our results show that an HFF diet fed to juvenile Iberian pigs produces a less oxidative skeletal muscle phenotype, similar to a detraining effect, and reduces the capacity to use lipid as fuel, even in the absence of insulin resistance and obesity.

## 1. Introduction

Pediatric non-alcoholic fatty liver disease (NAFLD) is the most common cause of chronic liver disease in children in developed countries, with a significant rise observed in countries undergoing epidemiological transition [1]. In the United States alone, approximately 83 million individuals are affected by NAFLD, with the prevalence predicted to increase 21% by the year 2030 [2]. NAFLD is characterized in the early stages by ectopic lipid accumulation in the liver (i.e., steatosis) in the absence of substantial alcohol intake [3]. Untreated NAFLD can progress to non-alcoholic steatohepatitis (NASH) with hepatocellular degeneration and inflammation, fibrosis, and eventually cirrhosis and liver failure [4].

Recently, several studies have reported an association between NAFLD and skeletal muscle loss and weakness in both aging and pediatric populations. A low skeletal muscle mass is associated with a greater risk of NAFLD, both independently and when combined with obesity [5,6,7,8,9,10]. Individuals with sarcopenia are 33% more likely to develop NAFLD, 56% more likely to experience significant fibrosis associated with NAFLD, and 142% more likely to develop NASH, independent of obesity [7]. Animal models of pediatric populations suggest that “Western-style diets” are likely to disrupt metabolism in skeletal muscle fibers in ways that may alter muscle fiber type differentiation (see Supplementary Table S1). Once muscle fiber type is established during childhood, it may be resistant to change in adulthood [11]. Although some studies indicate that intensive exercise training can drive shifts between fast-twitch and slow-twitch fiber types [11,12,13,14,15], most individuals are likely to undergo only minimal change in muscle fiber type after developmental differentiation. Since the hypercaloric “Western-style diet” contributes to the development of NAFLD and NASH [3,16] and changes in muscle fiber type can be expected to alter fuel utilization by skeletal muscle [12], developmental fiber type shifts induced by a hypercaloric diet early in life could have lifelong impacts on skeletal muscle quality, metabolic functionality, and overall health.

Most recent research on the association between NAFLD and skeletal muscle has operated on the notion that skeletal muscle weakness and loss precede and contribute to the development of NAFLD, as opposed to being consequences of the altered metabolic pathways seen in liver disease [5,6,7,8,9,10]. For example, patients with myotonic dystrophy, a genetic condition characterized by degeneration of muscle tissue and function, have an increased risk of developing NAFLD, with one study reporting that 44% of patients presented with both diseases concurrently [17,18]. The etiology of myotonic dystrophy is largely genetic, but little is understood about how lifestyle factors such as diet may contribute to the development and/or exacerbation of the condition. Recent evidence of a larger role for diet in myotonic dystrophy [19] leads to the question of whether diet-altered metabolic pathways may be an initiating factor in dysregulated skeletal muscle metabolism and health more generally. Several studies suggest that diet-based insulin resistance and inflammation may be the cause of concurrent skeletal muscle weakness and liver dyslipidemia, although there is limited evidence supporting this relationship [6,7,20]. To explore whether NAFLD precedes or accompanies a dysregulation of skeletal muscle composition and metabolism, we utilize a previously developed and characterized pediatric pig model of NAFLD induced by a high-fructose, high-fat (HFF) diet [21,22]. Juvenile Iberian pigs fed an HFF diet for 10 weeks developed steatosis, hepatocellular injury, and dysregulation of enterohepatic bile acid signaling similar to that observed in human children with NAFLD. We also found colonic hyperplasia and compositional shifts in intestinal microbial populations, which by promoting trimethylamine synthesis and possibly disrupting gut barrier function, may have caused damage to the liver and the brain cortex [21]. Animals also showed cholestasis and systemic choline deficiency despite adequate choline provision in the diet but did not have insulin resistance or obesity [21]. In this study, we utilize this model to investigate whether pediatric NAFLD is associated with changes in muscle phenotype that might affect fuel utilization and storage despite the absence of obesity and insulin resistance, potentially linking a “Western-style diet” to changes in muscle quality and functionality.

## 2. Materials and Methods

Approval for all experimental procedures was obtained from the Institutional Animal Care and Use Committee of California State University (#1611) before experiments began, and all procedures were in compliance with the National Research Council *Guide for the Care and Use of Laboratory Animals*. Two previous publications characterized the Iberian pig NAFLD phenotype in terms of liver and gut histology, biochemistry, blood, and metabolomics [21] and documented impairment of neurogenesis in the cerebral frontal cortex [22].

### 2.1. Animals and Experimental Design

As described previously [21], 13-day old male (M) and female (F) Iberian pigs (*n* = 28), were obtained from California Polytechnic State University’s Iberian Pig Research Colony after weaning and transported to a room with controlled temperature and a 12-h light-dark cycle. Animals were housed in 1.5 × 1.5 m pens in pairs, balanced for weight and sex, and given a volume of liquid feed comparable to the volume of milk typically consumed during lactation (45 mL · kg BW^−1^) at 6-h intervals. Pigs were randomized to receive one of the following liquid diets as detailed by Hernandez et al. [21] (g · kg body weight (BW)^−1^ ∙ day^−1^): (i) control (CON-N, *n* = 8, 4 M/4 F, 199.3 kcal metabolizable energy [ME], 0 g fructose, and 11.2 g fat); (ii) CON-P (*n* = 8, 5 M/3 F, CON-N diet + 6.2 × 10^4^ cfu ∙ mL^−1^ probiotics); (iii) high-fructose, high-fat (HFF-N, *n* = 6, 3 M/3 F, 314.8 kcal ME, 10 g fructose, and 20.6 g fat); or (iv) HFF-P (*n* = 6, 3 M/3 F, HFF-N diet + 6.2 × 10^4^ cfu ∙ mL^−1^ probiotics). The macronutrient composition and fructose and cholesterol concentrations of the HFF and HFF-P diets were based on the 5B4L diet used to produce NAFLD in Ossabaw pigs [23]. The probiotic blend was selected for its bile salt hydrolase activity observed in vivo [21] and its potential for hypocholesterolemic effects [24] and included *Bacillus amyloliquefaciens*, *Lactobacillus plantarum*, *Pediococcus pentosaceus*, and *Pediococcus acidilactici* (MultiBio 3PS; BiOWiSH Technologies, Cincinnati, OH, USA). Full details of the experimental diet are given in a previous report [21].

**Figure 1:**
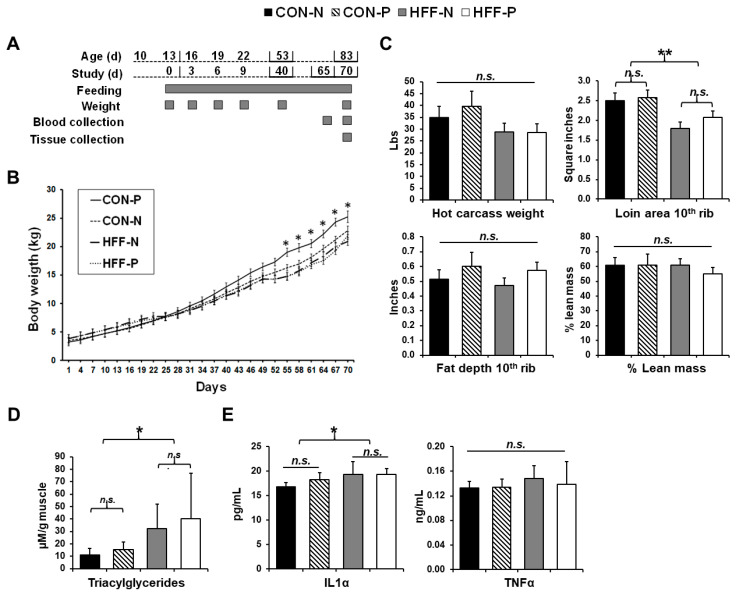
Lipid accumulation and inflammation in skeletal muscle of juvenile Iberian pigs fed high-fructose, high-fat diets (HFF) in the absence of overall changes in body composition. (**A**) Age of pigs related to study and feeding duration and weight, blood, and tissue collection points; (**B**) pig body weight (kg) throughout the duration of the study. A significant increase in the body weight of pigs fed a control diet with probiotic supplementation occurs beginning on day 55; (**C**) hot carcass weight (lbs), loin area at 10th rib (in^2^), and fat depth at 10th rib (in) were used to calculate lean mass percentage with the formula 100% × [8.588 + (0.465 × hot carcass weight) − (21.896 × 10th rib fat depth) + (3.005 × 10th rib loin muscle area)]/hot carcass weight to reveal no significant differences in lean mass or overall body composition between the groups; (**D**) triacylglycerol content (μM/g of muscle) of the longissimus dorsi muscle demonstrating a significant increase in lipid accumulation in HFF-fed pigs with a slight ameliorative effect observed with probiotic supplementation; (**E**) interleukin 1 alpha (IL-1α, pg/mL) and tumor necrosis factor-alpha (TNFα, ng/mL) content of the longissimus dorsi muscle with a significant increase in IL-1α in HFF-fed versus CON-fed pigs. CON—control; CON-N—control diet without probiotic supplementation; CON-P—control diet with probiotic supplementation; HFF-N—high-fructose, high-fat diet without probiotic supplementation; HFF—P-high-fructose, high-fat diet with probiotic supplementation; cm—centimeter; Mm—millimolar; g--gram; kg—kilogram; pg—picogram; ng—nanogram; mL—milliliter. Statistical significance between groups is indicated by * (*p* ≤ 0.05), ** (*p* ≤ 0.01). Non-significant differences between groups are indicated by “*n.s.*’’.

Pigs were weighed every three days throughout the study and the updated weights were used to set food rations for the next three-day period. On day 65, blood samples were collected from the jugular vein at 2-h post-feeding (Figure 1A). On d 70 (83 d of age), following an 8 h overnight fast, blood samples were collected from the left ventricle, and euthanasia was performed through an intramuscular injection of zolazepam and tiletamine (4 mg ∙ kg^−1^; Telazol; Zoetis, Parsippany, NJ) followed by intracardiac injection of pentobarbital sodium (0.4 mL · kg BW^−1^; Schering-Plough, Union, NJ, USA). Hot carcass weight (lbs), loin area at 10th rib (inches^2^), and fat depth at 10th rib (inches) were measured post-mortem and used to calculate lean mass percentage with the formula 100% × [8.588 + (0.465 × hot carcass weight)—(21.896 × 10th rib fat depth) + (3.005 × 10th rib loin muscle area)]/hot carcass weight.

Samples of longissimus dorsi muscle samples were obtained from 20 pigs immediately after euthanasia and dissected free of connective tissue. Samples for gene expression analysis were placed in 2 mL cryotubes and frozen by immersion in liquid nitrogen. Muscle samples for histology were placed on slices of cork, covered in optimum cutting temperature compound (Tissue-Tek Cryomold Standard; Sakura, Torrance, CA, USA), and frozen by immersion in isopentane suspended in liquid nitrogen. Samples were stored at −70 °C until analysis.

### 2.2. Triacylglycerol Content

Total triacylglycerol (TAG) in longissimus dorsi muscle was measured using previously described methods [25].

### 2.3. Cytokine Analyses

Tissue lysate homogenates were prepared for the muscle of pigs following a protocol by Sikalidis and Stipanuk [26]. Briefly, tissues were homogenized in cold TNESV lysis buffer supplemented with protease and phosphatase inhibitors (50 mmol/L Tris, pH 7.5, 1% [*v*:*v*] Nonidet P-40, 2 mmol/L EDTA, 150 mmol/L NaCl, and 10 mmol/L sodium orthovanadate), supplemented with 1 × PhosSTOP phosphatase inhibitor cocktail (Roche Applied Science, Penzberg, Germany) and 1 × Complete Protease Inhibitor Cocktail (Roche), to form 20% (*wt*:*v*) homogenates. Homogenates were centrifuged at 4 °C at 18,000× *g* for 20 min to obtain the soluble fraction (supernatant). Protein concentration was determined using the bicinchoninic acid (BCA) assay (Pierce) and equal amounts of total protein were loaded for ELISA analyses. For ELISA, 40 μg of total supernatant protein from each sample was loaded into each well. Analyses were conducted for porcine tumor necrosis factor-alpha (TNFα) and interleukin 1 alpha (IL-1α). Samples were run in duplicate on ELISA plates by Ray Biotech Inc., Norcross, GA following manufacturers’ protocol, with an intra-assay coefficient of variation (CV%) range of 1.6–6.4 and an inter-assay CV% range of 3.8–7.1.

### 2.4. Histological Parameters

Longissimus dorsi muscle samples were sectioned using a cryostat at a thickness of 10 μm and air-dried onto slides that had been pretreated with Vectabond (Vector Laboratories, Inc., Burlingame, CA, USA). The prepared slides were kept at −20 °C until staining. Oil red O (ORO) stain (Hartman-Leddon Co., Inc., Philadephia, PA, USA) was prepared according to Humason’s Animal Tissue Techniques [27]. Unfixed prepared skeletal muscle slides were rinsed in 60% isopropanol for 30 s, then stained in the ORO stain mixture for ten minutes. The slides were then rinsed in tap water for 5 min, mounted in glycerin jelly and viewed at magnifications between 10× and 400× total magnification.

Blocking solution for immunohistochemical staining was made by diluting goat serum (Invitrogen, Inc., Carlsbad, CA, USA) to 5% (*w*/*v*) in phosphate-buffered saline (PBS). Slides were stained concurrently with a primary stain solution containing myosin type I antibody (BAF8, dil. 1:500), myosin type IIA antibody (SC71, dil. 1:500), and myosin type IIB antibody (BFF3, dil. 1:500), in 5% goat serum in PBS. Primary antibodies for IHC were obtained from the Developmental Studies Hybridoma Bank, Iowa City, IA, USA. The secondary staining solution contained Alexa Fluor (AF) 350 (goat anti-mouse IgG2b, conjugates with BAF8, dil. 1:500, Invitrogen), AF488 (goat anti-mouse IgG1, conjugates with SC71, dil. 1:500, Invitrogen), and AF555 (goat anti-mouse IgM, conjugates with BFF3, dil. 1:500, Invitrogen) in 5% goat serum in PBS. Prepared skeletal muscle slides were blocked for an hour at room temperature (RT), rinsed in PBS three times, and then incubated overnight in the primary staining solution at 4 °C. The following day the slides were rinsed with PBS as above, incubated in the dark for an hour in the secondary staining solution, rinsed again with PBS, then mounted with Vectashield antifade reagent (Vector Laboratories, Inc., Burlingame, CA, USA).

Random images were taken of each stained slide at 200× or 400× total magnification by capturing an image in the top left corner, then moving diagonally down and to the right a fixed distance and taking another image until there were four images per sample. On small samples, the process was repeated starting in the bottom left corner and moving up and to the right once the bottom right corner was reached. ORO stain intensity was measured using Image J/FIJI after inverting the color lookup tables. Cells were counted by fiber type and presence or absence of ORO staining by manually counting all the cells in each field of view and dividing the cells stained by the total number of cells.

### 2.5. Western Blot Analyses

Thirty-five micrograms of muscle protein from each sample was separated by SDS-PAGE using a 10% gel for the protein kinase B (PKB) and ubiquitin-binding protein (p62) or 15% gel for the microtubule-associated protein light chain 3 (LC3) I/II. The proteins were then transferred to a polyvinylidene fluoride membrane (Thermo Fisher Scientific, Waltham, MA, USA) and blocked for 1 h at room temperature (RT) with 5% bovine serum albumin (VWR Life Science, Radnor, PA, USA) diluted in Tris-buffered saline [2.42 g Tris Base (RPI), 8 g NaCl (Thermo Fisher Scientific) and Tween 20 (0.1%; VWR Life Sciences)] (TBST). The membranes were incubated overnight at 4 °C with the primary antibody (dil. 1:1000, Cell Signaling, Danvers, MA, USA), washed the following day 3 × with PBST, and next incubated with the secondary antibody solution (goat anti-rabbit IgG, dil. 1:5000, Invitrogen) for 1 h at RT. An enhanced chemiluminescence kit (Amersham ECL Prime; GE Healthcare, Little Chalfont, UK) was used to develop the membranes, which were imaged on a ChemiDoc-It Imaging System (BIO-RAD, Hercules, CA, USA). Bands were quantified with Image Lab Software (Version 6.0.1; BIO-RAD).

### 2.6. Gene Expression

RNA was isolated with Tri Reagent (Molecular Research Center, Inc., Cincinnati, OH, USA) following the manufacturer’s protocol using bromochloropropane as the phase separation reagent. For RNA isolation, 50 to 100 mg of muscle tissue was immersed in 1.0 mL ice-cold Tri Reagent with ~10 1.6 mm stainless steel beads. The muscle was minced with fine dissecting scissors and then the tubes were shaken for 45 s in a FastPrep 120 bead homogenizer at top speed. Final RNA pellets were resuspended in 50 μL nuclease-free water and RNA concentration and quality were checked with a P300 NanoPhotometer (Implen GmbH, München, Germany.) (all A260/A280 ratios > 1.9). RNA was also visualized by electrophoresis on 1.1% agarose gels with SYBR Safe stain to ensure that any fragmentation was minimal.

DNAse treatment was performed on 10 μg of RNA from each sample using the Zymo RNA Clean and Concentrator 5 Kit (Zymo Research, Irvine, CA, USA), and the treated RNA was eluted in 20 μL of nuclease-free water. After DNAse treatment, cDNA was prepared from 1.5 μg of the resulting RNA using the Applied Biosystems high-capacity cDNA reverse transcription kit (Applied Biosystems, Waltham, MA, USA).

Real-time quantitative PCR (qPCR) was used to measure relative levels of mRNA encoding genes of interest. Primers targeting coding regions of each mRNA were designed using NCBI PrimerBlast and synthesized by Invitrogen (Supplementary Table S3). qPCR reactions were run in duplicate on a CFX96 Real-Time PCR System (BioRad Laboratories, Inc.) in 20 μL reactions using the ABI PowerUp SYBR Green Master Mix, with 1 μL of cDNA included in each reaction well. PCR reactions followed a thermal profile of 95 °C for 2 min, followed by 40 cycles of 95 °C for 15 s, 59 °C for 15 s, and 72 °C for 60 s, followed by melt curve analysis. Standard curves were based on a five-point serial dilution (1 to 256-fold) of a pool of combined cDNA from all 20 animals. Each qPCR plate included control reactions without cDNA and reactions containing RNA that did not undergo reverse transcription, all of which yielded negligible fluorescence signals. PCR efficiency for each plate was calculated as efficiency = 10^(−1/slope)^ − 1 where the slope is the slope of Cq vs. log(starting quantity). All qPCR efficiencies were between 93% and 114%, and the correlation coefficient (r^2^) for each standard curve was >0.98. The initial mRNA expression level for each gene of interest in each animal was normalized to the expression of the housekeeping gene, *TOP2B*, in that pig.

### 2.7. Metabolomics Analysis

A metabolomics assay capturing acylcarnitines was performed on plasma samples using protein precipitation extraction with ultra-performance liquid chromatography (UPLC) tandem quadrupole mass spectrometry, as described previously [21]. Briefly, 25 µL plasma was added to 1.5 mL Eppendorf tubes before being spiked with 20 µL isotopically labeled surrogates, followed by 750 µL chilled methanol. Samples were then vortexed 1 min prior to being centrifuged at 2000× *g* for 10 min. The supernatant was transferred to 1.5 mL high-performance liquid chromatography amber glass vials, dried by centrifugal vacuum evaporation, and reconstituted in 3:1 methanol:acetonitrile containing 100 nM of 1-cyclohexyl-ureido, 3-dodecanoic acid (Cayman Chemical Company, Ann Arbor, MI, USA). The reconstituted solution was vortexed 1 min and filtered at 0.1 µm by centrifugation at 2000× *g* for 10 min through PVDF Durapore membranes (Millipore, Billerica, MA, USA). Metabolites were separated using a Waters UPLC Acquity I-Class system (Waters, Milford, MA, USA) equipped with a 150 × 2.1 mm Atlantis HILIC column (Waters) interfaced with a 4000 QTRAP mass spectrometer (SCIEX, Framingham, MA, USA) operated in positive mode electrospray ionization and detected by multiple reaction monitoring. Metabolite intensities were normalized to values for internal standards added during the extraction, and to sample weight to account for small variations in starting tissue. General metabolites were expressed as peak areas under the curve.

### 2.8. Statistical Analyses

As described previously [21], body weight, loin area, fat depth, calculated lean mass, muscle triacylglycerol, gene and protein expression, histological measurements, cytokines, and serum biochemistry were analyzed using a mixed model two-way ANOVA in SAS 9.2 (SAS Institute, Cary, NC, USA), with pen as the experimental unit. The model considered diet × probiotic as a fixed effect and pen nested in diet × probiotic as a random effect. To account for interblot variability in the Western blot data, that analysis also included blot as a random effect. The presence of outliers and normality of the residuals was assessed in SAS, and the maximum likelihood method [28] was used to estimate the optimal value of the parameter used to power transform non-normally distributed parameters. Tukey test was utilized to correct for multiple comparisons. All data are represented as the least square mean ± standard deviation (SD) and results with a *p*-value less than or equal to 0.05 were considered significant.

Primer-E software (version 7; Primer-E Ltd., Plymouth, UK) was used to analyze the metabolomics data with a Euclidean distance matrix after log transformation into a normal distribution approximation. The null hypothesis of no difference between dietary groups was tested through a nonparametric permutational analysis of variance (PERMANOVA; Primer-E), using the same effects noted above, under a reduced model of type III sum of squares with 9999 permutations. Concentrations were cube-root transformed, scaled to unit variance, then underwent principal component analysis (PCA) with a two-dimensional scores plot to visualize group discrimination using the prcomp() function in the R Statistical Language. Component 1 and 2 PCA scores were plotted with overlays for diet and probiotic status. The group differences of component 1 were assessed using ANOVA, reporting the *p*-value, F statistic, and total R^2^. A two-way ANOVA with the aforementioned random and fixed effects was performed to identify the metabolites differentially expressed between diet groups.

## 3. Results

### 3.1. Liver Histology, Serum Biochemistry and Body Composition

Significant (*p* ≤ 0.05) interaction between diet and probiotics is reported and discussed for the CON-N, CON-P, HFF-N, or HFF-P groups; otherwise, results are discussed as a comparison between the diets (CON versus HFF) and/or effect of probiotic supplementation.

As described previously, over 80% of juvenile Iberian pigs fed the HFF diet for 10 consecutive weeks developed histopathological lesions consistent with NASH at 83 d of age, including steatosis, hepatocellular ballooning, Mallory hyaline, lobular inflammation, and necrosis [21]. In addition, 43% of CON and 17% of HFF-fed pigs developed steatosis without inflammation and/or cellular ballooning, and 57% of the CON group had no lesions in the liver. Further, HFF-fed pigs presented gut dysbiosis and hyperplasia that were positively correlated with the severity of the hepatic injury, as well as metabolic changes in liver, plasma, and colon digesta consistent with choline depletion and dysregulation of one-carbon metabolism [21,22]. Compared to CON, HFF-fed pigs had decreased cholesterol (*p* ≤ 0.01), and high and low-density lipoproteins (*p* ≤ 0.05), whereas triglycerides, non-esterified fatty acids, leptin, and pro-inflammatory cytokines TNF-α, IL-1α, and TNF-β did not differ across groups. Compared to non-probiotic animals, inclusion of probiotics in CON and HFF groups increased fasting insulin levels (*p* ≤ 0.05) and the homeostatic model assessment index (*p* ≤ 0.05), which is a quantitative measure of IR [21,22].

HFF diet had no effect on body weight gain and overall body composition based on calculations of lean mass percentage [21], whereas probiotic inclusion increased body weight in CON compared with all other groups (*p* ≤ 0.05; Figure 1B). Loin cross-sectional area at the 10th rib was decreased 24% in HFF-fed compared with CON-fed pigs (*p* ≤ 0.01), while there were no significant differences among groups in fat depth at the 10th rib or hot carcass weight (*p* > 0.05; Figure 1C).

### 3.2. Triacylglycerol Content

HFF diet increased ectopic lipid accumulation in skeletal muscle. Although there was no obesity among any of the groups, TAG content was increased 2.7-fold in the muscle of HFF-fed pigs compared with CON (*p* ≤ 0.05; Figure 1D). Probiotic supplementation had no significant effect on TAG content (*p* > 0.05).

### 3.3. Cytokine Analyses

HFF diet caused inflammation in skeletal muscle. Analysis of the IL-1α content of the muscle found increased levels in the HFF-fed pigs versus CON (*p* ≤ 0.05; Figure 1E), revealing an inflammatory response in the muscle of pigs receiving the HFF diet. Analysis of the muscle TNFα levels revealed no significant differences among groups (*p* > 0.05; Figure 1E).

### 3.4. Histological Parameters

**Figure 2:**
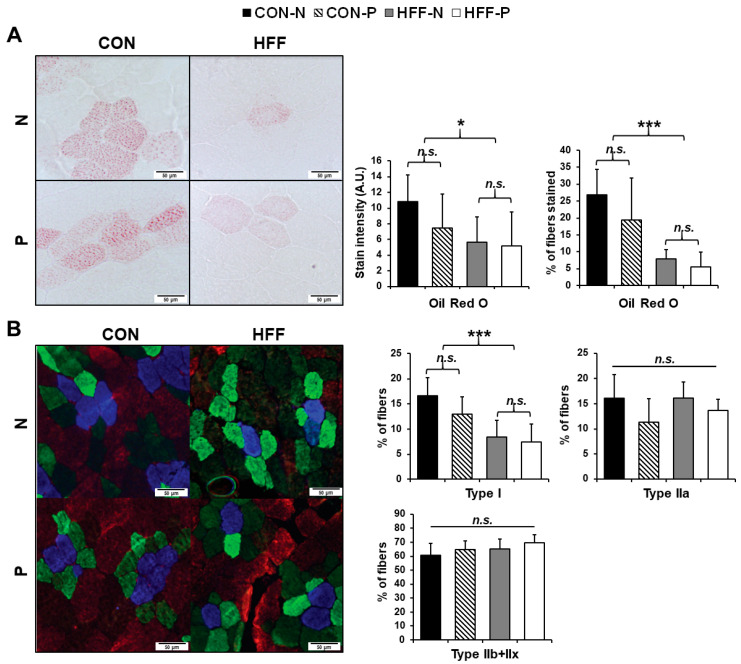
Effect of high-fructose, high-fat diet (HFF) on intramyocellular lipid (IMCL) content and skeletal muscle fiber type in juvenile Iberian pigs (*n* = 20). (**A**) Representative image of Oil red O (ORO) staining of longissimus dorsi muscle fibers [400× total magnification] with percentage of fibers staining with ORO (*n* = 3646) and ORO stain intensity (*n* = 918) (arbitrary units (AU)) demonstrating decreased IMCL in HFF-fed compared with CON-fed pigs; (**B**) representative image of immunohistochemical fiber typing of longissimus dorsi muscle [400× total magnification] with the total percentage of type I, type IIa, and type IIb/x fibers stained (*n* = 12682) indicating a decrease in type I slow oxidative fibers. Blue stained cells—Type I skeletal muscle fibers; green-stained cells—Type IIa; red-stained cells—Type IIb; unstained cells—Type IIx; CON—control; CON-N—control diet without probiotic supplementation; CON-P—control diet with probiotic supplementation; HFF-N—high-fructose, high-fat diet without probiotic supplementation; HFF—P-high-fructose, high-fat diet with probiotic supplementation. Statistical significance between groups is indicated by * (*p* ≤ 0.05), *** (*p* ≤ 0.001). Non-significant differences between groups are indicated by “*n.s.*’’.

Longissimus dorsi muscle of HFF-fed pigs demonstrated greater extramyocellular and decreased intramyocellular lipids. Oil Red O staining for lipid in longissimus dorsi muscle cells demonstrated significantly fewer stained cells (*p* ≤ 0.001; Figure 2A) and decreased ORO stain intensity in the stained cells (*p* ≤ 0.05; Figure 2A) of the HFF-fed versus CON-fed pigs. This decrease in intramyocellular lipid (IMCL) concentration suggests a decrease in lipid fuel utilization in the muscle cells of HFF-fed pigs. Qualitatively, greater deposits of extramyocellular lipids were observed in the HFF groups (Supplementary Figure S1) which, along with the higher overall TAG content in HFF-fed muscle, suggests greater ectopic lipid accumulation compared with CON groups.

HFF diet leads to a less oxidative skeletal muscle phenotype. There were significantly fewer type I slow oxidative muscle fibers in HFF-fed pigs as compared to CON (*p* ≤ 0.001; Figure 2B), matching the decrease in cells stained with ORO. Although probiotic effects on fiber type were not significant (*p* > 0.05), type I slow oxidative fibers appeared to be further decreased in the probiotic cohorts of each dietary group (Figure 2B). There were no significant differences between treatment groups in proportions of the other fiber types (type IIa fast oxidative, type IIx, type IIb, or hybrid fibers) (*p* > 0.05; Figure 2B).

### 3.5. Western Blot Analyses

**Figure 3:**
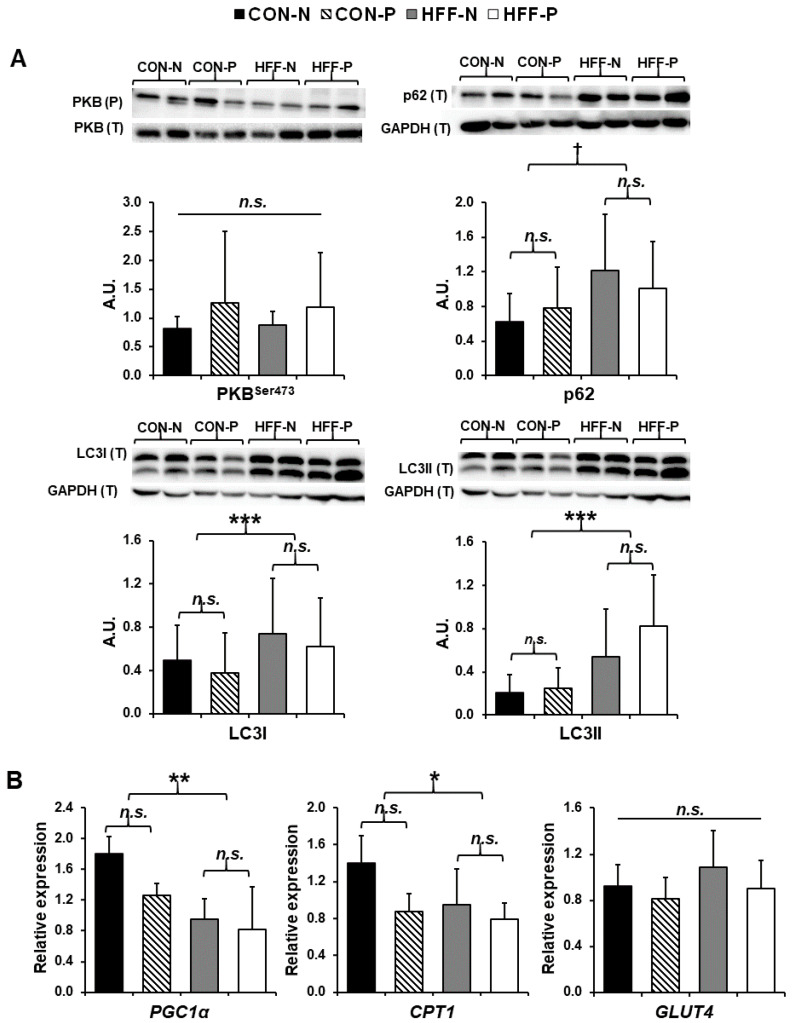
Increased autophagy and decreased lipid metabolism in skeletal muscle of juvenile Iberian pigs fed a high-fructose, high-fat diet (HFF) in the absence of insulin resistance. (**A**) Western blots of microtubule-associated protein 1 light chain 3 I (LC3I), microtubule-associated protein 1 light chain 3 II (LC3II), ubiquitin-binding protein (p62), and the ratio of phosphorylated to total protein kinase B (PKB Ser 473) in longissimus dorsi muscle indicating an increase in autophagy of HFF-fed versus CON-fed pigs with no change in insulin sensitivity; (**B**) gene expression of peroxisome proliferator-activated receptor gamma coactivator 1 alpha (PGC1α), carnitine palmitoyltransferase I (CPT1), and glucose transporter type 4 (GLUT4) in longissimus dorsi muscle as measured by qPCR demonstrating a significant decrease in genes encoding proteins imperative to lipid metabolism and β-oxidation. CON—control; CON-N—control diet without probiotic supplementation; CON-P—control diet with probiotic supplementation; HFF-N—high-fructose, high-fat diet without probiotic supplementation; HFF—P-high-fructose, high-fat diet with probiotic supplementation; qPCR—quantitative polymerase chain reaction. Statistical significance between groups is indicated by * (*p* ≤ 0.05), ** (*p* ≤ 0.01), and *** (*p* ≤ 0.001). Moderate trends indicated by † (*p* ≤ 0.1). Non-significant differences between groups are indicated by “*n.s.*’’.

HFF diet increased autophagy in skeletal muscle in the absence of insulin resistance. Skeletal muscle autophagy increased in the HFF-fed pigs compared with CON-fed pigs as evidenced by significant upregulation of LC3I (*p* ≤ 0.001; Figure 3A) and LC3II (*p* ≤ 0.001; Figure 3A), with a trend toward upregulation for p62 (*p* ≤ 0.1; Figure 3A). There was no evidence of insulin resistance in skeletal muscle as no significant differences were seen in the ratio of phosphorylated to total PKB (Ser 473) between any of the groups (*p* > 0.05; Figure 3A), although there was a trend toward increased phosphorylation of PKB with probiotic supplementation.

### 3.6. Gene Expression

HFF diet decreased markers of β-oxidation in skeletal muscle in the absence of insulin resistance. Compared to CON pigs, muscle from HFF-fed pigs had lower levels of mRNA encoding peroxisome proliferator-activated receptor-gamma coactivator 1 α (PGC1α) (*p* < 0.01) and carnitine palmitoyltransferase I (CPT1) (*p* ≤ 0.05), with no significant probiotic effect (Figure 3B). No significant difference in the expression of glucose transporter type 4 (GLUT4) was found between any of the groups (*p* > 0.05; Figure 3B).

### 3.7. Metabolomics Analysis

**Figure 4:**
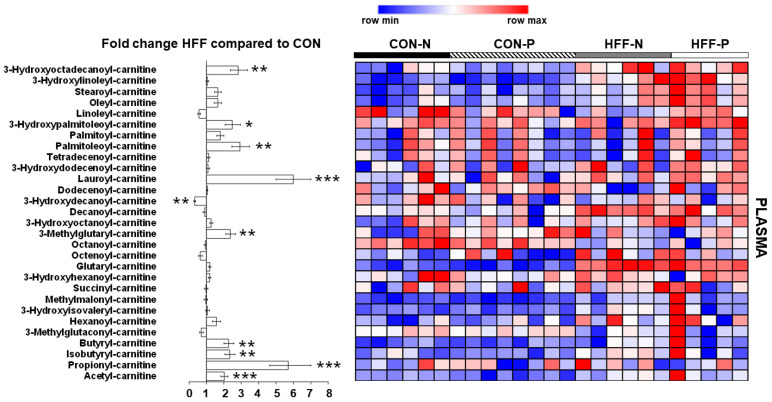
Effect of high-fructose, high-fat diet (HFF) on plasma metabolites related to dysregulated skeletal muscle composition and metabolism in juvenile Iberian pigs. Levels of several acylcarnitines are increased in plasma of HFF-fed versus CON-fed pigs, indicating dysregulated lipid metabolism. CON—control; CON-N—control diet without probiotic supplementation; CON-P—control diet with probiotic supplementation; HFF-N—high-fructose, high-fat diet without probiotic supplementation; HFF—P-high-fructose, high-fat diet with probiotic supplementation. Statistical significance between groups is indicated by * (*p* ≤ 0.05), ** (*p* ≤ 0.01), and *** (*p* ≤ 0.001).

HFF diet altered plasma metabolites related to dysregulated skeletal muscle composition and metabolism. Additionally, markers of dysregulated β-oxidation were altered in HFF-fed pigs compared with CON, including increases in multiple acylcarnitines (*p* ≤ 0.05; Figure 4) which are intermediate metabolites in lipid oxidation pathways.

## 4. Discussion

In the work presented here, we investigated the connection between “Western diet”-induced pediatric NAFLD and altered skeletal muscle fuel utilization and lipid storage. We used a model in which juvenile Iberian pigs fed a hypercaloric “Western-style diet”, with and without probiotic supplementation, developed NAFLD and NASH [21]. This model is distinct from many other animal models in that pigs fed the high-fructose, high-fat (HFF) diet developed liver pathology in the absence of (or prior to) obesity or insulin resistance [16,21,29,30]. Here, we show that the skeletal muscle of HFF-fed pigs displayed ectopic extracellular lipid accumulation, inflammation, and autophagy. Additionally, the HFF diet led to a reduction in intramyocellular lipids (IMCL) and differentiation toward a less oxidative phenotype in skeletal muscle. Along with alterations in plasma metabolites, these changes suggest a dysregulation of skeletal muscle energy metabolism similar to a detraining effect.

The “Western-style diet”, characterized by an excess consumption of calories from both fructose and fat, has been shown to produce deleterious effects on metabolic health leading to obesity, insulin resistance, systemic inflammation, and decreased capacity for physical work, as well as the ectopic lipid accumulation seen in NAFLD and NASH [16,29,30,31,32]. Over the last 20 years, there has been a dramatic rise in obesity and associated metabolic conditions in children globally [33]. Likewise, obesity rates among American children 2–19 years old have risen by 8.5% since 1994 according to the 2015–2016 National Health and Nutrition Examination Survey (NHANES) [34]. The increase in overnutrition and obesity has been accompanied by a doubling in pediatric NAFLD incidence over two decades, thus making NAFLD the most common cause of pediatric chronic liver disease in the United States [1]. Recently, there has been debate over whether the skeletal muscle abnormalities that accompany NAFLD are a consequence of the disease or a causal factor contributing to its development [7]. We, therefore, utilized the HFF diet to induce NAFLD and NASH in juvenile Iberian pigs and investigate dietary effects on skeletal muscle during development. We cannot rule out the possibility that additional metabolic pathologies induced by the HFF-diet may play a role in the observed changes in muscle phenotype in this study. The short duration of HFF feeding and absence of measurable obesity or insulin resistance suggest that NAFLD and/or direct dietary effects on muscle cells are important factors, but other secondary diet-induced changes may also be involved.

We found that HFF feeding produced ectopic lipid accumulation in the longissimus dorsi muscle, as indicated by the significant increase in triacylglycerol content and qualitative increase in extramyocellular lipids (EMCL), in agreement with similar studies in mice [35]. The accompanying reduction in intramyocellular lipid (IMCL) staining suggests a decrease in lipid fuel utilization in the muscle cells of HFF-fed pigs. Results of the metabolomic analysis are consistent with this shift, as increases in numerous plasma acylcarnitines indicate incomplete fatty acid oxidation in skeletal muscle of HFF-fed pigs [36]. The drop in IMCL contrasts with increases in IMCL seen in most previous studies investigating skeletal muscle responses to a high-fructose diet, a high-fat diet, or a combination HFF diet (see Supplementary Tables S1 and S2). This difference may reflect the fact that obesity and insulin resistance accompany liver pathology in most other studies, but are absent in our model, at least over the timeframe of the study. Increased IMCL has been found to correlate with insulin resistance when corrected for physical activity [37]; however, we also observed no significant difference in activity levels between the dietary groups, as described in a previous report [22].

Within skeletal muscle, type I (slow oxidative) cells have the greatest capacity to oxidize lipid fuels. Corresponding to the reduction in IMCL, HFF-fed pigs had fewer slow oxidative type I skeletal muscle fibers and more glycolytic type IIb and/or type IIx muscle fibers, cells that primarily utilize glucose for fuel. The shift in fiber types suggests a decrease in skeletal muscle oxidative capacity in HFF-fed pigs, similarly to what is observed in muscle atrophy or detraining, which causes shifts to generally faster and more glycolytic fiber types [38]. This shift in muscle fiber type and concomitant shifts in fuel utilization may have a substantial impact on the capacity for endurance-type physical activity [39]. Although many studies have demonstrated training- or detraining-induced shifts between subtypes of type II skeletal muscle fibers, it has been difficult to produce changes between type I and type II fibers through exercise, particularly in humans [11,12,13,14,15,38]. Prolonged, intense exercise programs are required to produce even modest changes in the proportion of type I and type II fibers [40,41], hence such changes would rarely be relevant in countering the influence of poor diets. Therefore, the decrease in type I fibers and oxidative capacity in juvenile skeletal muscle seen here is likely to be permanent, with concerning implications for life-long health and fitness. The initial change in fiber-type composition may be followed by a long-term decrease in muscle mass and strength. While the reduced loin cross-sectional area observed in HFF-fed pigs may be explained by the trend toward smaller body size, the decrease in plasma creatinine reported previously [21] might suggest a tendency toward lower muscle growth in the HFF-fed pigs.

Reductions in gene expression of *PGC1α* and *CPT1b* in the HFF-fed pigs provide additional evidence for a decrease in mitochondrial biogenesis and decreased capacity for mitochondrial fatty acid oxidation, respectively [42,43]. The decreased skeletal muscle oxidative capacity indicated by these changes suggests a loss of metabolic flexibility—the ability of a tissue or organism to adapt to changing energy conditions such as energy demand or availability of different fuel types [13]. This encompasses transitions between fasting and fed states and physical activity. The ability of skeletal muscle to store fuel appropriately and switch between fatty acid oxidation, glucose oxidation, and glycolysis is crucial not only for maintaining skeletal muscle function in varying conditions but also for whole-body energy balance [44]. A decrease in skeletal muscle oxidative capacity directly translates to a decrease in metabolic flexibility, which is likely to have systemic, potentially long-term effects. The measurements of increased levels of multiple acylcarnitines in plasma suggest that incomplete fatty acid oxidation is occurring in the muscle, which may reflect a prolonged mismatch between lipid supply and lipid oxidation [45].

NAFLD has also been associated with skeletal muscle weakness [5,6,7,8,9,10]. In the present study, we saw no significant difference in lean muscle mass percentage between any of the dietary groups and no change in physical activity, as described in a previous report [22]. However, HFF-fed pigs exhibit increases in autophagy biomarkers, microtubule-associated protein light chain 3 I and II (LC3I/II), and a trend toward increased ubiquitin-binding protein (p62). Increases in these markers of autophagy have been linked to muscle atrophy, along with decreases in gene expression of *PGC1α*, a protein important in mitochondrial biogenesis. The HFF-fed pigs also showed increases in the proinflammatory cytokine, interleukin 1 alpha (IL-1α), as typically seen in muscle catabolism [46]. Western blotting revealed no significant differences in the PKB content of skeletal muscle between any of the dietary groups, increases in which are known to inhibit autophagy [47]. In addition, we previously reported that HFF-fed pigs had lower levels of plasma creatinine [21], which have been linked to decreased muscle mass and utilization [36].

Previous studies have demonstrated correlations between choline deficiency, NAFLD, and skeletal muscle aberrations, but most studies have examined the causal role of a choline-deficient diet in the etiology of liver disease rather than the potential for liver disease to produce choline deficiency [48,49]. A previous report indicated that Iberian pigs fed an HFF, choline-sufficient diet develop a systemic choline deficiency [21]. Choline deficiency has been linked to hypermetabolism, weight loss, and improved insulin sensitivity [50,51] and might contribute to muscle atrophy through a decrease in the neurotransmitter, acetylcholine, which can cause deficiencies in neuromuscular transmission and contribute to muscle autophagy [47,49,52,53]. Although we did not measure muscle strength in the present study, an association between NAFLD and muscle weakness has long been recognized in elderly populations who may be experiencing age-related sarcopenia and has recently also been found in pediatric populations [8]. This weakness could result from or be exacerbated by skeletal muscle changes similar to those demonstrated in our study. Together, these results suggest a dysregulation of skeletal muscle metabolism and a trend toward muscle atrophy in the absence of measurable changes in lean muscle mass.

One strength of the present study is the selection of Iberian pigs as a model for pediatric NAFLD. The majority of the experimental metabolic studies investigating skeletal muscle responses to a high-fat and/or high-fructose diet have used rodents, which have a notably different metabolism than humans due to their body size and energy requirements [54]. While rodent models are cost-effective, it is unclear how well outcomes from those models translate to humans. However, animal models are necessary for long-term randomized studies of diets that may have deleterious health consequences [55,56,57]. The use of a pig model allows us to bridge the gap between experimental rodent studies and observational human studies and take advantage of a longer developmental scale as compared to rodents [58,59,60]. Many dietary studies on rodents have involved a dietary challenge that begins in adolescence and continues through adulthood, but this design does not allow for differentiation between developmental and post-developmental effects of the diet (see Supplementary Table S1). Utilizing a juvenile pig model provides time to fully mimic the effects of a “Western-style diet” comparable to developmental stages in human children and more time in each developmental stage of liver disease. With the rising prevalence of NAFLD in children [1], such models are increasingly vital to observing disease progression and possible long-term effects of developmental changes.

A unique aspect of the present study was the use of probiotics to supplement both the HFF and CON diets. In recent years, probiotics have increased in popularity as dietary supplements in the U.S., with adult probiotic use quadrupling from 2007 to 2012, according to the 2012 National Health Interview Survey [61]. Numerous studies have suggested that probiotics can ameliorate negative effects of hypercaloric diets such as weight gain, insulin resistance, and NAFLD in both human [62,63] and animal models [64,65]. Despite the dramatic rise in popularity of probiotics, much is still unknown about how probiotics might affect or interact with various diets and disease states. In this experiment, probiotic supplementation was unable to mitigate the pathological effects of the hypercaloric HFF diet. Probiotics appeared to intensify some effects of the HFF diet, such as the decrease in muscle oxidative capacity and increased body weight of control-fed pigs, but probiotic effects were mostly non-significant or inconsistent. It is important to note that probiotic supplements vary widely [66], and this study investigated only one combination of probiotic microbial strains in the context of one experimental diet. Nonetheless, it is possible that some probiotic strains may amplify the effects of a hypercaloric diet, potentially by increasing absorption of excess nutrients or altering the regulation of nutrient absorption. More studies are needed to confirm the safety and efficacy of the wide variety of probiotic supplements in individuals with metabolic disorders.

We should also note that our study examined only one muscle, the longissimus dorsi. Pigs have been employed in a number of studies using hypercaloric diets to produce liver disease, obesity, and/or insulin resistance, and these studies have used a variety of pig strains and skeletal muscles including soleus, plantaris, biceps femoris, and longissimus dorsi [31,67,68,69]. The variability of study systems may be an advantage; if each model reproduces human disease processes imperfectly or represents disease processes in a subset of human patients, then it is important to use multiple animal models. The longissimus dorsi is appropriate for this study as it contains a mix of muscle fiber types. However, analysis of dietary effects on muscles with a greater proportion of slow oxidative cells, such as soleus or tibialis cranialis, would also be interesting.

This study was a collaborative opportunity with an experimental design focused on investigating liver and gut pathology in the Iberian pig model; our muscle studies were thus limited by the original study design in terms of diet, duration, and sample size. In practice, the hypercaloric “Western-style” diet varies widely, and no single experimental diet can represent the full spectrum of hypercaloric diets as they vary among humans or within an individual over time [70]. Because the pigs were housed in pairs controlled for sex and the pens constituted the experimental unit, we were unable to test for differences between sexes. Additionally, our study was limited by the lack of access to dual-energy X-ray absorptiometry (DEXA), magnetic resonance imaging (MRI), or computed tomography (CT), imaging technologies that would have permitted a more thorough assessment of body composition and lean muscle mass. Future studies would benefit from utilizing these imaging technologies to obtain more accurate and comprehensive measurements of lean muscle mass. The study also relied on several indirect measurements of muscle lipid oxidative capacity and anabolic/catabolic state. For instance, *CPT1b* mRNA levels are an indirect indicator of the capacity for lipid oxidation in muscle. While CPT1 protein levels play an important role in setting the maximum rate of long-chain fatty acid oxidation [43,71], measurements of the protein levels or activities of additional enzymes would provide a more comprehensive picture of the ability to process lipid fuels. Likewise, measurements of physical activity tolerance in vivo and measurements of muscle strength would be beneficial. Additionally, measurement of the cross-sectional areas of whole muscles would provide a more thorough assessment of differences in muscle mass.

## 5. Conclusions

The present study tested a hypercaloric “Western-style diet” high in both fructose and fat that causes juvenile Iberian pigs to develop NAFLD and NASH. We found that the HFF diet led to the accumulation of ectopic extracellular lipid in skeletal muscle, despite the absence of changes in overall body composition and obesity. The HFF diet also increased skeletal muscle inflammation and autophagy without inducing insulin resistance. The HFF diet lowered intramyocellular lipid storage and led to a less oxidative phenotype in skeletal muscle, with altered plasma metabolites suggesting dysregulation of skeletal muscle energy metabolism, similar to a detraining effect. Probiotics were tested in combination with the HFF diet and appeared to exacerbate some of these changes, such as the shifts in muscle fiber type and intramyocellular lipid storage, but results relating to probiotics were mostly inconclusive.

## Supplementary Materials

The following are available online at https://www.mdpi.com/article/10.3390/nu13124195/s1, Figure S1: Representative Oil red O-stained image of longissimus dorsi muscle showing increased extramyocellular lipid in juvenile Iberian pigs fed a high-fructose, high-fat diet [100× total magnification]. Table S1: Research studies investigating the effects of a Western-style diet on skeletal muscle in juvenile animal models. Table S2: Research studies investigating the effects of a Western-style diet on skeletal muscle in adult animal models and humans. Table S3: Primer sequences utilized in qPCR analysis of gene expression in the skeletal muscle of juvenile Iberian pigs.

## Abbreviations

NAFLD	Non-alcoholic fatty liver disease
CON	Control
HFF	High-fructose high-fat
NASH	Non-alcoholic steatohepatitis
M	Male
F	Female
BW	Body weight
CON-N	Control
ME	Metabolizable energy
CON-P	Control with probiotics
HFF-N	High-fructose high-fat
HFF-P	High-fructose high-fat with probiotics
BCA	Bicinchoninic acid
TNFα	Tumor necrosis factor alpha
IL-1α	Interleukin 1 alpha
PKB	Protein kinase B
LC3	Microtubule-associated protein light chain 3
p62	Ubiquitin-binding protein
RT	Room temperature
TBST	Tris-buffered saline with tween
UPLC	Ultra-performance liquid chromatography
PBS	Phosphate-buffered saline
ORO	Oil red O
AF	Alexa Fluor
TAG	Triacylglycerol
SE	Standard error
PCA	Principal component analysis
IMCL	Intramyocellular lipid
EMCL	Extramyocellular lipid
CPT1	Carnitine palmitoyltransferase I
PGC1α	Peroxisome proliferator-activated receptor gamma coactivator 1 alpha
GLUT4	Glucose transporter type 4
TOP2B	DNA topoisomerase 2β
