# Supplementary material for: High-Fructose, High-Fat Diet Alters Muscle Composition and Fuel Utilization in a Juvenile Iberian Pig Model of Non-Alcoholic Fatty Liver Disease
Source: Nutrients. 2021 Nov 23;13(12):4195. doi: 10.3390/nu13124195 (PMC8705774; doi:10.3390/nu13124195)
Supplement: Supplementary file 1 [file nutrients-13-04195-s001.zip › nutrients-1449449-supplementary.pdf]

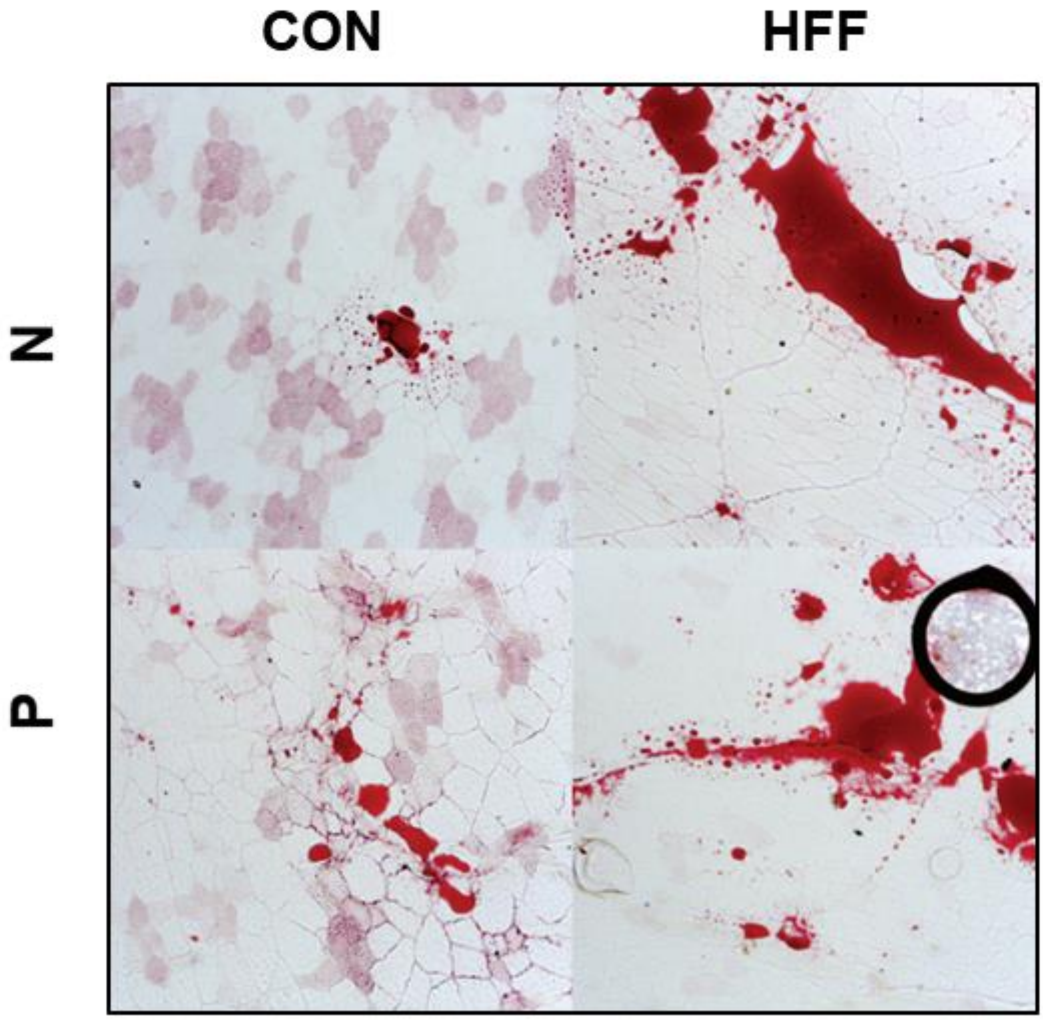

Figure S1. Representative Oil red O stained image of longissimus dorsi muscle showing increased extramyocellular lipid in juvenile Iberian pigs fed a high-fructose, high-fat diet [100X total magnification]. CON-N--control diet without probiotic supplementation; CON-P--control diet with probiotic supplementation; HFF-N--high-fructose, high-fat diet without probiotic supplementation; HFF--P-high-fructose, high-fat diet with probiotic supplementation.

Table S1. Research studies investigating the effects of a Western-style diet on skeletal muscle in juvenile animal models.

Author	Subjects	Age Start	Age End	Feeding	Outcome
Hua et al. 2017 [35]	C57 BLK/6J mice (male)	4 wks	10 wks	*Ad libitum*: HF (60% kcal fat)	HF: Increased BW, TG, EMCL, slightly increased IMCL, Increase ratio of EMCL:IMCL, type I -> II shift (type I lumbar muscles)
Baena et al. 2016 [72]	Sprague-Dawley rats (female)	8 wks	16 wks	*Ad libitum*: CON (solid feed; 4502±229 kcal/d) HFr (CON+10% w/v Fru liquid; 7277±735 kcal/d) HGlu (CON+10% w/v Glu liquid; 7489±354 kcal/d)	HFr and HGlu: Increased liquid consumption and reduced solid food consumption. Non-sig changes in BW. Higher plasma TG and hyperinsulinemia. HFr: Less reduction in plasma NEFA following insulin administration. HFr and HGlu on SkM (gastroc): Unimpaired p-AKT and total AS160 following insulin administration. NC in fusion capability of GLUT4 vesicles to plasma membrane. HGlu on SkM (gastroc): Increased expression of GLUT4 in plasma membrane. HFr SkM (gastroc): Reduced expression of GLUT4 in plasma membrane.
Crescenzo et al. 2013 [73]	Sprague-Dawley rats (male)	90 d (12.9 wks)	146 d (20.9 wks)	CON (4.11 kcal/g, 60.4% kcal CHO [0% Fru, 45.3% starch, 15.1% sugars], 10.6% kcal fat) HFr (4.11 kcal/g, 60.4% kcal CHO [30% Fru, 22.8% starch, 7.6% sugars], 10.6% kcal fat)	HFr: Increased plasma NEFA. Higher body energy w/ similar energy intakes, indicative of increased efficiency of metabolism. Hyperinsulinemia with no sig difference in plasma glu. Lower RMR values by wk 4. HFr on SkM (hind leg muscle): Increased total lipids, TG, and ceramide. NC in p-AKT:AKT ratio, but lower p-AKT when normalized to plasma insulin levels. Higher cytochrome c:actin ratio and increased state 3 mito respiration. Increased mito mass and coupling.
Crescenzo et al. 2015 [29]	Sprague-Dawley rats (male)	90 d (12.9 wks)	104 d (14.8 wks)	LF (59.8 kcal/d, 67.9% kcal CHO [67.9% starch], 11.3% kcal fat) HF (59.8 kcal/d, 36.6% kcal CHO [36.6% starch], 42.5% kcal fat) HFF (59.8 kcal/d, 36.6% kcal CHO [25% kcal Fru, 11.6% starch], 42.5% kcal fat)	HF and HFF: Higher body energy, lipids, and energetic efficiency. Decreased net energy expenditure. HF: Higher plasma NEFA vs. CON. HFF: Higher plasma TG and NEFA vs. CON and HF. Increased HOMA index. Increased glu and insulin response. HF and HFF on SKM (hind leg muscles): Lowered oligomycin state 4 respiration. Increased mito coupling. Decreased ANT and increased UCP3. HF on SKM (hind leg muscles): Higher total lipids, TG, and ceramide vs. CON. HFF on SkM (hind leg muscles): Higher total lipids, TG, and ceramide vs. CON and HF. Decreased insulin sensitivity. Decreased p-AKT following insulin administration. NC in state 3, state 4, RCR or cytochrome c:actin ratio of mito.

Higashiura et al. 1999 [74]	Sprague-Dawley rats (male)	6 wk	12 wk	*Ad libitum*: HFr (60% kcal Fru)	HFr: Insulin resistance, lower BW, higher BP, decreased type I, increased type IIa, non-sig increased type IIb (sol) (Myosin ATPase stain)
Thomas et al. 2014 [75]	C57 BLK/6J mice (male)	4 wk	7 wk	*Ad libitum*: HF (60% kcal fat)	HF: Insulin resistance, higher BW, type IIb -> IIa/x shift (TA). Non-sig. increased type IIa, NC type I, IIx (sol), increased SDH density in type I and IIa fibers (sol), NC SDH density (TA), lower capillary density, NC in IMCL, palmitate oxidation, oxidative phosphorylation proteins, exercise capacity, FAT/CD36
Olver et al. 2018 [30]	Ossabaw miniature pigs (male and female)	3.5 mo	6 mo	CON (3.03 kcal/g, 71% kcal CHO, 10.5% kcal fat) HFFC (4.14 kcal/g, 40.8% kcal CHO [17.8% Fru], 43% kcal fat, 2% kcal C)	HFFC: Increased BW and induced obesity at wk 6. Increased plasma NEFA w/ NC in plasma TG. Increased plasma glu w/ NC in plasma insulin or HOMA-IR. Decreased physical activity. HFFC on SkM (triceps): Non-sig increase p-AKT:AKT ratio following insulin administration. Increased total lipids and SFAs, w/ trend towards increased MUFA. Lowered ratio of n3:n6 fatty acids.
Ozkan & Yakan 2019 [76]	Wistar albino rats (male)	12 wks (3 mo)	24 wks (6 mo)	*Ad libitum*: CON (2600 kcal, 54.7% kcal CHO, 3.15% kcal fat) HF (3600 kcal, 39.7% kcal CHO, 13.5% kcal fat) HFr (2600 kcal, 54.7% kcal CHO [27% Fru], 3.15% kcal fat) HSuc (2600 kcal, 54.7% kcal CHO [27% Suc], 3.15% kcal fat)	All Diets on SkM (gluteus maximus): NC in PPARα and ChREBP gene expression. NC in PPARα protein content. HFr: Higher BW as of wk 3 and average higher intake of kcals/wk. Higher plasma TG and glucose. HSuc: Lowest plasma glucose. HFr on SkM (left gluteus maximus): Increased LXRα and SREBP-1c gene expression. Increased SREBP-1c and ChREBP protein content. Overall: SREBP-1c levels were found to be correlated with plasma glucose and TG levels. LXRa levels were found to be correlated with plasma glucose levels.
Cabot et al. 2012 [77]	Wistar rats (male)	8 wks	12 wks	*Ad libitum*: HF (similar to a cafeteria diet)	HF: Increased IMCL, mitochondria (sol)
Wang et al. 2019 [78]	Wistar rats (male)	6 wks	10, 14, 18, 22, and 26 wks	*Ad libitum*: CON (81% kcal CHO [10% Suc, 0% Fru], 4% kcal fat [17.4% SFA]) HFF (47% kcal CHO [0% Suc, 25% Fru], 35% kcal fat [44.2% SFA])	HFF: Lower dietary intakes at wk 4 and higher intakes at wks 12 and 16. Increased BW. Glu intolerance. NC in serum TG and decreased serum FFAs. HFF on SkM (sol, tib, EDL): Slightly lower but sig. muscle weight. HFF on SKM (sol): Increased total DAG at 4 wks (positively correlated with glu intolerance). Increased total ceramides at 4 and 12 wks (not correlated with glu intolerance). Increased gene expression of CD36, PLIN2, and ATGL at wk 16. HFF on SkM (gastroc): Lower muscle weight. Increased TG and FFA at 12 wks (positively correlated with glu

					intolerance). NC in total phospholipids or cardiolipin. Increased total SFA and MUFA and decreased PUFA. Decreased unsaturation index of FAs and SCD1 gene expression. NC in protein expression of FATCD36, FATP1, ACSL1, PPARα, PPARγ, SREBP-1c, mCPT-1, PGC-1a, or ACC. Increased gene expression of FABP3. Decreased FAS protein content. NC in mito membrane potential, respiration, or fluidity. NC in citrate synthase or β-HAD. NC in gene expression of CDS1. Decrease in gene expression of CLS, but NC in protein content.
DeNies et al. 2014 [79]	C57 BLK/6J mice (male and female)	3 wk	55 wk	*Ad libitum*: HF (60% kcal fat)	HF: Higher BW, lower type I, non-sig higher I/IIa hybrids (only in male), NC MHCI (sol)
De Stefanis et al. 2017 [80]	C57 BLK/6J mice (male)	5 wk	33 wk	*Ad libitum*: HFr (15% kcal Fru in water) HGlu (15% kcal Glu in water)	HFr: Insulin resistance, increased BW, TG (muscle), IMCL, CSA, LC3B-II, non-sig beclin-1, IL-6 (muscle), NC in ox/glyc fibers, (SDH) (gastroc), PAX7, myogenin, p63, fibrinogen, SOCS3 HGlu: Insulin resistance, increased BW, TG (muscle), non-sig IMCL, CSA, beclin-1, LC3B-II, IL-6 (muscle and plasma), NC in ox/glyc fibers, (SDH) (gastroc), PAX7, myogenin, p63, fibrinogen, SOCS3
Roseno et al. 2015 [81]	C57 BLK/6J mice (male)	8 wk	20 wk	*Ad libitum*: HF (60% kcal fat), for 3 wks or 12 wks	HF - 3 wks: Higher BW, non-sig insulin, NC glucose (plasma), myostatin HF - 12 wks: Higher BW, insulin, NC glucose (plasma), higher myostatin
Ferrer-Martínez et al. 2006 [82]	Wistar rats (male)	~10-15 wk (200-225g)	~14-19 wk (4 wk)	*Ad libitum*: HSuc (50% kcal Suc) HF (33.9% kcal fat)	HSuc: Insulin resistance, NC BW, higher fed TAG (plasma), non-sig lower FAT, FATP1, GLUT1 (sol), GLUT4, LPL (sol), PDK4, HK2 (sol), non-sig higher ACC1, GFAT1 (sol), LPL (gastroc), HK2 (gastroc), higher GLUT1 (gastroc), GFAT1 (gastroc), NC GK, GP HF: Insulin resistance,higher BW, fed TAG (plasma), non-sig lower FAT, FATP1, GLUT4 (sol), LPL, PDK4 (gastroc), non-sig higher GLUT1 (sol), GFAT1 (sol), HK2 (sol), PDK4 (sol), GLUT4 (gastroc), ACC1 (gastroc), higher ACC1 (sol), GLUT1 (gastroc), GFAT1 (gastroc), HK2 (gastroc), NC GK, GP
Vaisy et al. 2010 [83]	Wistar rats (male)	~10-15 wk (200-225g)	~22-27 wk (12 wk)	*Ad libitum*: HSuc (48% kcal Suc)	HSuc: Insulin resistance, higher BW, IMCL, NC UCP3
Lee et al. 2016 [84]	Sprague-Dawley rats (male)	~ 4-5 wks (95-	~12-13 wks	*Ad libitum*:	HF/Sat and HF/PUFA: NC in BW. Increased plasma FFA

		100 g BW)		CON (61.8% kcal CHO, 15.7% kcal fat)<br>HF/Sat (24.3% kcal CHO, 52.8% kcal fat [primarily SFA])<br>HF/PUFA (24.3% kcal CHO, 52.8% kcal fat [primarily PUFA])	HF/Sat: Increased fasting plasma insulin and HOMA-IR.<br>HF/PUFA: Decreased fasting plasma insulin and HOMA-IR.<br>HF/Sat and HF/PUFA on SkM (vastus lateralis): Increased TG and DAG. NC in ceramide content.<br>HF/Sat on SkM (vastus lateralis): Increased SFA in total TG. Increased SDC1 protein expression and enzyme activity.<br>HF/PUFA on SkM (vastus lateralis): Increased unsaturated FA and decreased SFA in total TG.
Rosholt et al. 1994 [85]	Sprague-Dawley rats (male)	~ 5 wks (100-125 g)	~ 8 wks	*Ad libitum*:<br>HC (1910±51 kcal, 68% kcal CHO, 10% kcal fat)<br>HF (1700±37 kcal, 20% kcal CHO, 60% kcal fat)<br>Then treated with insulin, exercise, or nothing (control) before termination.	HF: Lower overall energy intake. Higher plasma FFA. NC in plasma glu or insulin.<br>HF on SkM (gastroc): NC in muscle glycogen content and non-responsive to insulin treatment. Higher TG, not affected by insulin or exercise. NC in KpNPPase enzyme activity. NC in glu uptake/Vmax w/ insulin and exercise treatments. Increased GLUT4 protein content w/ insulin or exercise treatment only.<br>HC on SkM (gastroc): NC in muscle glycogen content but increased w/ insulin treatment. NC in KpNPPase enzyme activity. Increased glu uptake/Vmax w/ insulin and exercise treatments. Increased GLUT4 protein content w/ insulin or exercise treatment only.
Li et al. 2008 [86]	Wistar rats (male)	4 wks (80-100 g)	10 wks	*Ad libitum*:<br>CON (59% kcal CHO, 12% kcal fat)<br>HFr (60% kcal CHO [?% Fru], 12% kcal fat)	HFr: NC in BW or fasting blood glu. Higher fasting plasma insulin. Decreased GIR and MCR.<br>HFr on SkM (hind limb): NC in total AKT or PKCγ protein content but decreases in both p-AKT and p-PKCγ. NC in total GLUT4 content but decreased plasma membrane GLUT4.
Benetti et al. 2018 [87]	C57 BLK/6J mice (male)	5 wks	21 wks	*Ad libitum*:<br>HFGluFr (45% kcal fat, 19.24% kcal Fru, 15.75% kcal Glu)	HFGluFr: Increased BW, HOMA-IR, lower glucose tolerance, higher LDL-c, NC TC, TG (plasma), higher p-IRS-1, lower p-Akt, increased nuclear p65, TNFα, IMCL (unquantified), SCAP expression, active SREBP1c, RAGE
Freitas et al. 2009 [88]	Wistar rats (male)	13 wk	23 wk	*Ad libitum*:<br>HFSuc (32% kcal fat, 48% kcal CHO)	HFSuc: Higher BW, NC oxidative vs. glycolytic fibers, capillary density
Marotta et al. 2004 [89]	Wistar rats (male)	~11-16 wk (200-225g)	~15-20 wk (4 wk)	*Ad libitum*:<br>HFL (33.9% kcal by weight lard)<br>HFO (33.9% kcal by weight high	HFL: Insulin resistance, higher BW, epididymal fat, fed TG, fed FFA, FATP-1<br>HFO: Insulin resistance, higher BW, epididymal fat, fed TG, fed FFA, FATP-1

				oleic sunflower oil)<br>HFS (33.9% kcal by weight sunflower oil)	HFS: Insulin resistance, higher BW, epididymal fat, fed TG, fed FFA, FATP-1
Chicco et al. 2003 [90]	Wistar rats (male)	~7-13 wk (175-190g)	~10-43 wk (3, 15, or 30 wk)	*Ad libitum*:<br>HSuc (63% kcal Suc by weight), for 3 wks, 15 wks, or 20 wks	HSuc - 3 wk: Insulin resistance, NC BW, higher plasma TG, FFA, higher LCACoA, NC glycogen, GS, PDHK and PDH activity, G6P, TG (muscle)<br>HSuc - 15 wk: Insulin resistance (sig higher than 3 wk), NC BW, higher plasma TG (sig higher than 3 wk), FFA (sig higher than 3 wk), lower glycogen, GS, PDHK and PDH activity, G6P, higher TG (muscle)<br>HSuc - 30 wk: Insulin resistance (sig higher than 3 wk), higher BW, higher plasma TG (sig higher than 3 wk), FFA (sig higher than 3 wk), lower glycogen, GS, PDHK and PDH activity, G6P, higher TG (muscle)
Simi et al. 1991 [91]	Wistar rats (male)	~5-8 wk (~160g)	~17-20 wk (12 wk)	*Ad libitum*:<br>HF (48% kcal by weight lard)	HF: NC BW, higher CS (quad) and HAD (quad and sol) activities, NC HK and PFK activities, NC glycogen (muscle), lower glycogen breakdown, higher FFA,

Weeks (wks), days (d), skeletal muscle (SkM), extramyocellular lipids (EMCL), intramyocellular lipids (IMCL), body weight (BW), mitochondria (Mito), low-fat diet (LF), high-fat diet (HF), ethanol (Eth), high-fat, high-fructose diet (HFF), high-fat, high-fructose, high-cholesterol diet (HFFC), high-fat, high-sucrose diet (HFSuc), high-fructose diet (HFr), high-sucrose diet (HSuc), high-glucose diet (HGlu), glucose (Glu), area under the curve (AUC), standard chow/control diet (CON), cholesterol (C), high-density lipoprotein cholesterol (HDL-c), low-density lipoprotein cholesterol (LDL-c), total cholesterol (TC), triglycerides (TG), diacylglycerols (DAG), saturated fatty acids (SFA), monounsaturated fatty acids (MUFA), polyunsaturated fatty acids (PUFA), free fatty acids (FFA), fasting serum glucose (FSG), homeostatic model assessment of insulin resistance (HOMA-IR), glucose transporter type 4 (GLUT-4), nonesterified fatty acids (NEFA), resting metabolic rate (RMR), uncoupling protein 3 (UCP3), soleus muscle (Sol), plantaris muscle (Pln), gastrocnemius muscle (Gastroc), tibialis (Tib), extensor digitorum longus (EDL), tibialis anterior muscle (TA), gastrocnemius soleus tibialis anterior and quadriceps (Hind leg muscles), peroxisome proliferator-activated receptor α (PPARα), liver X receptor alpha (LXRα), carbohydrate response element binding protein (ChREBP), stearoyl-CoA desaturase (SDC-1), cardiolipin synthase (CLS), fatty acid binding protein 3 (FABP3), fatty acid translocase CD36 (FATCD36), glycerol kinase (GK), Glycogen phosphorylase (GP), protein kinase B (AKT), phosphorylated protein kinase B (p-AKT), AKT substrate of 160 kDA (AS160), long chain acyl CoA (LCACoA), insulin receptor substrate 1 (IRS-1), phosphorylated insulin receptor substrate 1 (p-IRS-1), fatty acid transport protein 1 (FATP-1), glucose-6-phosphate (G6P), pyruvate dehydrogenase (PDH), pyruvate dehydrogenase kinase (PDHK), 3-hydroxyacyl-CoA dehydrogenase (HAD), hexokinase (HK), phosphofructokinase (PFK), mitochondrial respiratory control ratio (RCR), adenine nucleotide translocase (ANT).

Table S1 References

35. Hua, N.; Takahashi, H.; Yee, G.M.; Kitajima, Y.; Katagiri, S.; Kojima, M.; Anzai, K.; Eguchi, Y.; Hamilton, J.A. Influence of muscle fiber type composition on early fat accumulation under high-fat diet challenge. *PLoS ONE* **2017**, *12*, e0182430, doi:10.1371/journal.pone.0182430.
72. Baena, M.; Sangüesa, G.; Dávalos, A.; Latasa, M.-J.; Sala-Vila, A.; Sánchez, R.M.; Roglans, N.; Laguna, J.C.; Alegret, M. Fructose, but not glucose, impairs insulin signaling in the three major insulin-sensitive tissues. *Sci Rep* **2016**, *6*, 26149, doi:10.1038/srep26149.
73. Crescenzo, R.; Bianco, F.; Coppola, P.; Mazzoli, A.; Cigliano, L.; Liverini, G.; Iossa, S. Increased skeletal muscle mitochondrial efficiency in rats with fructose-induced alteration in glucose tolerance. *Br J Nutr* **2013**, *110*, 1996–2003, doi:10.1017/S0007114513001566.
29. Crescenzo, R.; Bianco, F.; Coppola, P.; Mazzoli, A.; Cigliano, L.; Liverini, G.; Iossa, S. The effect of high-fat–high-fructose diet on skeletal muscle mitochondrial energetics in adult rats. *Eur J Nutr* **2015**, *54*, 183–192, doi:10.1007/s00394-014-0699-7.
74. Higashiura, K. Alteration of muscle fiber composition linking to insulin resistance and hypertension in fructose-fed rats. *American Journal of Hypertension* **1999**, *12*, 596–602, doi:10.1016/S0895-7061(99)00025-4.
75. Thomas, M.M.; Trajcevski, K.E.; Coleman, S.K.; Jiang, M.; Di Michele, J.; O'Neill, H.M.; Lally, J.S.; Steinberg, G.R.; Hawke, T.J. Early oxidative shifts in mouse skeletal muscle morphology with high-fat diet consumption do not lead to functional improvements. *Physiol Rep* **2014**, *2*, e12149, doi:10.14814/phy2.12149.
30. Olver, T.D.; Grunewald, Z.I.; Jurrissen, T.J.; MacPherson, R.E.K.; LeBlanc, P.J.; Schnurbusch, T.R.; Czajkowski, A.M.; Laughlin, M.H.; Rector, R.S.; Bender, S.B.; et al. Microvascular insulin resistance in skeletal muscle and brain occurs early in the development of juvenile obesity in pigs. *American Journal of Physiology-Regulatory, Integrative and Comparative Physiology* **2018**, *314*, R252–R264, doi:10.1152/ajpregu.00213.2017.
76. Ozkan, H.; Yakan, A. Dietary high calories from sunflower oil, sucrose and fructose sources alters lipogenic genes expression levels in liver and skeletal muscle in rats. *Annals of Hepatology* **2019**, *18*, 715–724, doi:10.1016/j.aohep.2019.03.013.
77. Cabot, C.; Pouillot, K.; Roy, S.; Romero, M.; Vilà, R.; Grasa, M.; Esteve, M.; Fernández-López, J.A.; Alemany, M.; Remesar, X. Effect of cafeteria diet feeding on soleus intramyocellular lipid of Wistar rats. *J Endocrinol Metab* **2012**, doi:10.4021/jem63w.
78. Wang, Y.; Fouret, G.; Bonafos, B.; Blachnio-Zabielska, A.; Leroy, T.; Crouzier, D.; Barea, B.; Gaillet, S.; Moro, C.; Lecomte, J.; et al. Long-term follow-up of muscle lipid accumulation, mitochondrial activity and oxidative stress and their relationship with impaired glucose homeostasis in high fat high fructose diet-fed rats. *The J of Nutr Biochem* **2019**, *64*, 182–197, doi:10.1016/j.jnutbio.2018.10.021.
79. DeNies, M.S.; Johnson, J.; Maliphol, A.B.; Bruno, M.; Kim, A.; Rizvi, A.; Rustici, K.; Medler, S. Diet-induced obesity alters skeletal muscle fiber types of male but not female mice. *Physiol Rep* **2014**, *2*, e00204, doi:10.1002/phy2.204.
80. De Stefanis, D.; Mastrocola, R.; Nigro, D.; Costelli, P.; Aragno, M. Effects of chronic sugar consumption on lipid accumulation and autophagy in the skeletal muscle. *Eur J Nutr* **2017**, *56*, 363–373, doi:10.1007/s00394-015-1086-8.
81. Roseno, S.L.; Davis, P.R.; Bollinger, L.M.; Powell, J.J.S.; Witczak, C.A.; Brault, J.J. Short-term, high-fat diet accelerates disuse atrophy and protein degradation in a muscle-specific manner in mice. *Nutrition and Metab* **2015**, 12, 39, doi:10.1186/s12986-015-0037-y.
82. Ferrer-Martínez, A.; Marotta, M.; Turini, M.; Mace, K, Gomez-Foix, A.M. Effect of sucrose and saturated-fat diets on mRNA levels of genes limiting muscle fatty acid and glucose supply in rats. *Lipids* **2006**, 41, 55-62, doi:10.1007/s11745-006-5070-1
83. Vaisy, M.; Szlufcik, K.; Maris, M.; De Bock, K.; Hesselink, M.K.C.; Eijnde, B.O.; Schrauwen, P.; Hespel, P. Hyperglycemic diet and training alter insulin sensitivity, intramyocellular lipid content but not UCP3 protein expression in rat skeletal muscles. *Int J Mol Med* **2010**, *25*, doi:10.3892/ijmm_00000421.
84. Lee, J.S.; Pinnamaneni, S.K.; Eo, S.J.; Cho, I.H.; Pyo, J.H.; Kim, C.K.; Sinclair, A.J.; Febbraio, M.A.; Watt, M.J. Saturated, but not n-6 polyunsaturated, fatty acids induce insulin resistance: role of intramuscular accumulation of lipid metabolites. *J Appl Physiol* **2006**, *100*, 1467–1474, doi:10.1152/japplphysiol.01438.2005.
85. Rosholt, M.N.; King, P.A.; Horton, E.S. High-fat diet reduces glucose transporter responses to both insulin and exercise. *Am J Physiol-Regulatory, Integr and Comp Physiol* **1994**, *266*, R95–R101, doi:10.1152/ajpregu.1994.266.1.R95.
86. Li, P.; Koike, T.; Qin, B.; Kubota, M.; Kawata, Y.; Jia, Y.; Oshida, Y. A High-fructose diet impairs Akt and PKCζ phosphorylation and GLUT4 translocation in rat skeletal muscle. *Horm Metab Res* **2008**, *40*, 528–532, doi:10.1055/s-2008-1073162.
87. Benetti, E.; Mastrocola, R.; Chiazza, F.; Nigro, D.; D'Antona, G.; Bordano, V.; Fantozzi, R.; Aragno, M.; Collino, M.; Minetto, M.A. Effects of vitamin D on insulin resistance and myosteatosis in diet-induced obese mice. *PLoS ONE* **2018**, *13*, e0189707, doi:10.1371/journal.pone.0189707.
88. Freitas, I.F.; Gomes, J.J.;Sabino, J.P.J. Histochemical responses of rats exercised in two weekly frequencies and ingesting standard or hypercaloric diet. *Biol Sport* **2009**, *26(1)*, 71-85
89. Marotta, M.; Ferrer-Martínez, A.; Parnau, J.; Turini, M.; Macé, K.; Gómez Foix, A.M. Fiber type- and fatty acid composition-dependent effects of high-fat diets on rat muscle triacylglyceride and fatty acid transporter protein-1 content. *Metabolism* **2004**, *53*, 1032–1036, doi:10.1016/j.metabol.2004.03.011.
90. Chicco, A.; D'Alessandro, M.E.; Karabatas, L.; Pastorale, C.; Basabe, J.C.; Lombardo, Y.B. Muscle lipid metabolism and insulin secretion are altered in insulin-resistant rats fed a high sucrose diet. *The J of Nutrition* **2003**, *133*, 127–133, doi:10.1093/jn/133.1.127.
91. Simi, B.; Sempore, B.; Mayet, M.H.; Favier, R.J. Additive effects of training and high-fat diet on energy metabolism during exercise. *J Appl Physiol* **1991**, *71*, 197–203, doi:10.1152/jappl.1991.71.1.197.

Table S2. Research studies investigating the effects of a Western-style diet on skeletal muscle in adult animal models and humans.

Author	Subjects	Age Start	Age End	Feeding	Outcome
Clark et al. 2011 [67]	Ossabaw minipigs (male)	-	(24 wk study)	*Ad libitum*: HFr (20% kcal Fr) HFFC (20% kcal Fr, 46% kcal fat, 2% kcal C)	HFr: Higher BW, CSA (pln, biceps, sol), non-sig higher type IIb/x, lower type I (pln, biceps, sol), non-sig lower MHCI (pln), non-sig higher MHCIIb (pln), NC IMCL HFFC: Higher BW, IMCL, TG (blood), CSA (pln, biceps), lower CSA (sol) non-sig higher type IIb/x, lower type I (pln, biceps, sol), higher type IIa (biceps), decreased MHC1 (pln), non-sig higher MHCIIb (pln)
Hsu et al. 2017 [31]	Taiwan Lee-Sung miniature pigs (castrated males)	2 y	3 y	LF (restricted to ~2700 kcal/d, 66.4% kcal CHO [0% Fru], 17.8% kcal fat) HFF (*ad libitum* ~5200 kcal/d, 39.2% kcal CHO [17.8% Fru], 45% kcal fat)	HFF: Higher weight gain and obesity. Increased serum TG, NEFA, insulin. NC in serum glucose. Increased HOMA-IR. HFF on SkM (sol): Decreased p-AKT. Insulin resistance.
Hyatt et al. 2016 [92]	Rhesus monkeys (male)	~12 yr	~14 yr (2 yr)	*Ad libitum*: 2 meals/day HFS (42.3% kcal fat, 27% Suc)	HFS: Increased BW, type I, decreased type IIa/x (sol), NC in pln or EDL (western blot), decreased PGC-1a in EDL, non-sig decrease in pln, non-sig increase in sol, NC GLUT4
Rodriguez et al. 2015 [93]	C57/Bl6J mice (female)	12 wks	32 wks	*Ad libitum*: CON (67% kcal CHO [4% Suc], 10% kcal fat) HF (35% kcal CHO [17% Suc], 45% kcal fat) HF+Pom (same as HF) HF+GT (same as HF) Actual intakes not mentioned	All HF Diets: Higher BW and obesity. NC in total FoxO3a. NC in mRNA of IL-6 or MCP-1. NC in MAPK pathway. HF on SkM (gastroc): Decreased p-AKT with NC in mTOR. Increased mRNA and decreased phosphorylation of FoxO3a and FoxO1. Increased MAFbx, MURF1, p62, BNIP3L, and BNIP3. Non-sig increases in LC3b and Gabarapl-1.
Song et al. 2013 [94]	C57BL/6 J mice (male)	14-15 wks	30-31 wks	*Ad libitum*: CON (66% kcal CHO, 10% kcal fat) HF (20% kcal CHO, 59% kcal fat) HFr (70% kcal CHO [35% Fru], 9% kcal fat) Actual intakes not mentioned	HF and HFr: Higher BW, plasma TG, and plasma FFA. Glucose intolerance (more sig in HFr). HF and HFr on SkM (quad): Increased TG, LCACoA, and expression of FATCD36. Increased expression of COx, CS, β-HAD, COX-1, and CPT-1 enzymes. Increase in PGC-1α protein. Increased expression of SREBP-1c and FAS genes.
Deldicque et al. 2010 [95]	Humans (male)	~20 yr	~20 yr (6 wk)	Energy requirement-based on:	HF: Higher BW, glucose intolerance, higher IMCL, NC FFA (plasma), DAG (muscle), ceramides, phospholipids, phospho-PKB

				HF (50% kcal fat), hypercaloric (+30% kcal)	
Seyssel et al. 2016 [96]	Humans (male, healthy, high-risk for T2DM, first-degree relative of T2DM patient)	(not mentioned)	(7 d each w/ 4-5 wk washout)	RCT Crossover: CON (55% kcal CHO, 30% kcal fat) HFr (CON +3.5 g Fru/kg fat-free mass [+35% kcal] per d)	HFr on SkM (vastus lateralis): Increased lipid content and SREBP-1c mRNA expression. Sig shift in energy fuel substrates to CHO oxidation measured via IC. Downregulation of genes of ACADVL, ACADS, HADHA, HADHB, and ACAA2 (enzymes of β-oxidation). Downregulation of genes of CPT1B and CPT2 (needed for fat to enter mitochondria). Decreased MLYCD and L41 mRNA, UCP3 protein, and genes of respiratory enzymes (i.e. cytochrome c).
Osterberg et al. 2015 [63]	Humans (male)	18-30 yr	18-30 yr (4 wk)	Energy requirement-based on: HF (55% kcal fat), hypercaloric (+35% kcal)	HF: Higher body fat % and lean mass, non-sig BW, NC endotoxin, LBP, sCD14, hsCRP, higher TNFα, non-sig IL6, NC insulin sensitivity, fatty acid oxidation (muscle), pyruvate oxidation
Surowska et al. 2019 [97]	Humans (male and female)	~22 yr	~22 yr (6 d each)	Energy requirement-based on RCT w/ 4-8 wk washout: Hypercaloric (+50% kcal) LP-HF+HSuc (5% kcal protein, 25% kcal fat, 34% kcal Suc) HP-LF+HSuc (20% kcal protein, 10% kcal fat, 34% kcal Suc)	LP-HF: Higher BW, fasting and fed plasma fructose, lactate (sig higher than HP-LF), uric acid (sig higher than HP-LF), TG, NEFA, insulin, higher fasting glucose, higher IMCL (sig higher than HP-LF), NC fasting glucagon or IGF-1 HP-LF: Higher BW, fasting and fed plasma fructose, lactate, uric acid, TG, NEFA, insulin, higher fasting glucose, higher IMCL, NC fasting glucagon or IGF-1
Van Proeyen et al. 2011 [98]	Humans (male)	~21 yr	~21 yr (6 wk)	Normal energy intake-based on: HF (50% kcal fat), hypercaloric (>30% kcal)	HF: Higher BW, IMCL, exercise-induced IMCL breakdown, decreased exercise-induced glycogen breakdown, NC AMPKα, ACCβ, pAMPK, p-ACCβ, PDK4 expression, NC FFA

Weeks (wks), days (d), year (yr), treatment (Tx), significant (Sig), no change (NC), indirect calorimetry (IC), type 2 diabetes mellitus (T2DM), skeletal muscle (SkM), carbohydrate (CHO), extra-myocellular lipids (EMCL), intramyocellular lipids (IMCL), cholesterol (C), high-density lipoprotein cholesterol (HDL-c), low-density lipoprotein cholesterol (LDL-c), body weight (BW), low-fat diet (LF), high-fat diet (HF), high-saturated fat diet (HF/Sat), high-polyunsaturated fat diet (HF/PUFA), ethanol (Eth), high-fat high-fructose diet (HFF), high-fructose diet (HFr), high fat high sucrose diet (HFS), high-fat + pomegranate extract diet (HF+Pom), high-fat + green tea extract diet (HF+GT), high-carbohydrate diet (HC), fructose (Fru), sucrose (Suc), free fatty acids (FFA), normal control diet (CON), randomized cross-over trial (RCT), triglycerides (TG), total triglycerides (TTG), diacylglycerols (DAG), fasting serum glucose (FSG), homeostatic model assessment of insulin resistance (HOMA-IR), glucose transporter type 4 (GLUT-4), nonesterified fatty acids (NEFA), resting metabolic rate (RMR), uncoupling protein 3 (UCP3), protein kinase B (AKT), phosphorylated protein kinase B (p-AKT), stearoyl-CoA desaturase-1 (SDC1), soleus muscle (Sol), plantaris muscle (Pln), gastrocnemius muscle (Gastroc), quadriceps muscle (Quad), extensor digitorum longus muscle (EDL), tibialis anterior muscle (TA), forkhead box O3a (FoxO3a), forkhead box O1 (FoxO1), muscle atrophy F-box (MAFbx), muscle ring finger-1 (MURF1), ubiquitin-binding protein (p62), BCL2 Interacting Protein 3 Like (BNIP3L), BCL2 Interacting Protein 3 (BNIP3), Microtubule Associated Protein 1 Light Chain 3 Beta (LC3b), GABA Type A Receptor Associated

Protein Like 1 (Gabarapl-1), interleukin 6 (IL-6), Monocyte Chemoattractant Protein-1 (MCP-1), Mitogen-Activated Protein Kinase (MAPK), long chain acyl CoA (LCACoA), fatty acid translocase CD36 (FATCD36), cytochrome oxidase (COx), citrate synthase (CS), β-hydroxyacyl CoA dehydrogenase (β-HAD), carnitine palmitoyltransferase-1 (CPT-1), proliferator-activated receptor coactivator (PGC-1α), cyclo-oxygenase-1 (COX-1), murine sterol regulatory element-binding protein-1c (SREBP-1c), lipogenic enzyme fatty acid synthase (FAS), lipopolysaccharide binding protein (LBP), acyl-CoA dehydrogenase very long chain (ACADVL), acyl-CoA dehydrogenase C2 to C3 short chain (ACADS), hydroxyacyl-CoA dehydrogenase/3-ketoacyl-CoA thiolase/enoyl-CoA hydratase alpha and beta subunit (HADHA and HADHB), acetyl-CoA acyltransferase 2 (ACAA2), carnitine palmitoyltransferase 1B (CPT1B), carnitine palmitoyltransferase 2 (CPT2), malonyl-CoA decarboxylase (MLYCD), uncoupling protein 3 (UCP3), acetyl-CoA carboxylase (ACCβ), 5' AMP-activated protein kinase (AMPK), pyruvate dehydrogenase kinase 4 (PDK4).

Table S2 References

67. Clark, B.A.; Alloosh, M.; Wenzel, J.W.; Sturek, M.; Kostrominova, T.Y. Effect of diet-induced obesity and metabolic syndrome on skeletal muscles of Ossabaw miniature swine. *American Journal of Physiology-Endocrinology and Metabolism* **2011**, *300*, E848–E857, doi:10.1152/ajpendo.00534.2010.
31. Hsu, M.-C.; Wang, M.-E.; Jiang, Y.-F.; Liu, H.-C.; Chen, Y.-C.; Chiu, C.-H. Long-term feeding of high-fat plus high-fructose diet induces isolated impaired glucose tolerance and skeletal muscle insulin resistance in miniature pigs. *Diabetol Metab Syndr* **2017**, *9*, 81, doi:10.1186/s13098-017-0281-6.
92. Hyatt, J.-P.K.; Nguyen, L.; Hall, A.E.; Huber, A.M.; Kocan, J.C.; Mattison, J.A.; de Cabo, R.; LaRocque, J.R.; Talmadge, R.J. Muscle-specific myosin heavy chain shifts in response to a long-term high fat/high sugar diet and resveratrol treatment in nonhuman primates. *Front. Physiol.* **2016**, *7*, doi:10.3389/fphys.2016.00077.
93. Rodriguez, J.; Gilson, H.; Jamart, C.; Naslain, D.; Pierre, N.; Deldicque, L.; Francaux, M. Pomegranate and green tea extracts protect against ER stress induced by a high-fat diet in skeletal muscle of mice. *Eur J Nutr* **2015**, *54*, 377–389, doi:10.1007/s00394-014-0717-9.
94. Song, G.-Y.; Ren, L.-P.; Chen, S.-C.; Wang, C.; Liu, N.; Wei, L.-M.; Li, F.; Sun, W.; Peng, L.-B.; Tang, Y. Similar changes in muscle lipid metabolism are induced by chronic high-fructose feeding and high-fat feeding in C57BL/J6 mice. *Clin Exp Pharmacol Physiol* **2012**, *39*, 1011–1018, doi:10.1111/1440-1681.12017.
95. Deldicque, L.; Van Proeyen, K.; Francaux, M.; Hespel, P. The unfolded protein response in human skeletal muscle is not involved in the onset of glucose tolerance impairment induced by a fat-rich diet. *Eur J Appl Physiol* **2011**, *111*, 1553–1558, doi :10.1007/s00421-010-1783-1.
96. Seyssel, K.; Meugnier, E.; Lê, K.-A.; Durand, C.; Disse, E.; Blond, E.; Pays, L.; Nataf, S.; Brozek, J.; Vidal, H.; et al. Fructose overfeeding in first-degree relatives of type 2 diabetic patients impacts energy metabolism and mitochondrial functions in skeletal muscle. *Mol. Nutr. Food Res.* **2016**, *60*, 2691–2699, doi:10.1002/mnfr.201600407.
63. Osterberg, K.L.; Boutagy, N.E.; McMillan, R.P.; Stevens, J.R.; Frisard, M.I.; Kavanaugh, J.W.; Davy, B.M.; Davy, K.P.; Hulver, M.W. Probiotic supplementation attenuates increases in body mass and fat mass during high-fat diet in healthy young adults: probiotic supplementation during high-fat diet. *Obesity* **2015**, *23*, 2364–2370, doi:10.1002/oby.21230.
97. Surowska, A.; Jegatheesan, P.; Campos, V.; Marques, A.-S.; Egli, L.; Cros, J.; Rosset, R.; Lecoultre, V.; Kreis, R.; Boesch, C.; et al. Effects of dietary protein and fat content on intrahepatocellular and intramyocellular lipids during a 6-day hypercaloric, high sucrose diet: a randomized controlled trial in normal weight healthy subjects. *Nutrients* **2019**, *11*, 209, doi:10.3390/nu11010209.
98. Van Proeyen, K.; Szlufcik, K.; Nielens, H.; Deldicque, L.; Van Dyck, R.; Ramaekers, M.; Hespel, P. High-fat diet overrules the effects of training on fiber-specific intramyocellular lipid utilization during exercise. *Journal of Applied Physiology* **2011**, *111*, 108–116, doi:10.1152/japplphysiol.01459.2010.

Table S3. Primer sequences utilized in qPCR analysis of gene expression in the skeletal muscle of juvenile Iberian pigs.

Accession Number	Gene	Protein	Primer Sequence	Product Size (bp)
NM_213963.2	*PPARGC1A*	PPARG coactivator 1 alpha	F: AACCAGGACTCTGTATGGACTG<br>R: GTTCAGGAAGATCTGGGCAAAG	80
NM_001007191.1	*CPT1B*	Carnitine palmitoyltransferase 1B	F: CTCTGGACGAGGAGTCTCAC<br>R: CACCTGTTGTAGCAGTTGCC	97
NM_001128433.1	*GLUT4/ SLC2A4*	GLUT4 / Solute carrier family 2 member 4	F: AGTGGCTGGGAAGGAAGAAG<br>R: GGAACCGTCCAAGAATGAGC	120
NM_001258386.1	*TOP2B*	Topoisomerase (DNA) II beta	F: GCTGGTCCTGAAGATGATGC<br>R: TACGCTGTCTCCGATCTTCC	106

[1]Accession number is the unique identification number for the messenger RNA (mRNA) sequence for which primers were designed. Sequences were retrieved from the RefSeq database of the National Center for Biotechnology Information.
